# Supplementary material for: Pathological characterization of female reproductive organs prior to miscarriage induced by Zika virus infection in the pregnant common marmoset
Source: Microbiol Spectr. 2025 Feb 25;13(4):e02282-24. doi: 10.1128/spectrum.02282-24 (PMC11960083; doi:10.1128/spectrum.02282-24)
Supplement: Figure S1 — Double immunofluorescence staining of ZIKV NS1 and fibroblast markers in the basal layer of endometrium. [file spectrum.02282-24-s0001.pdf]

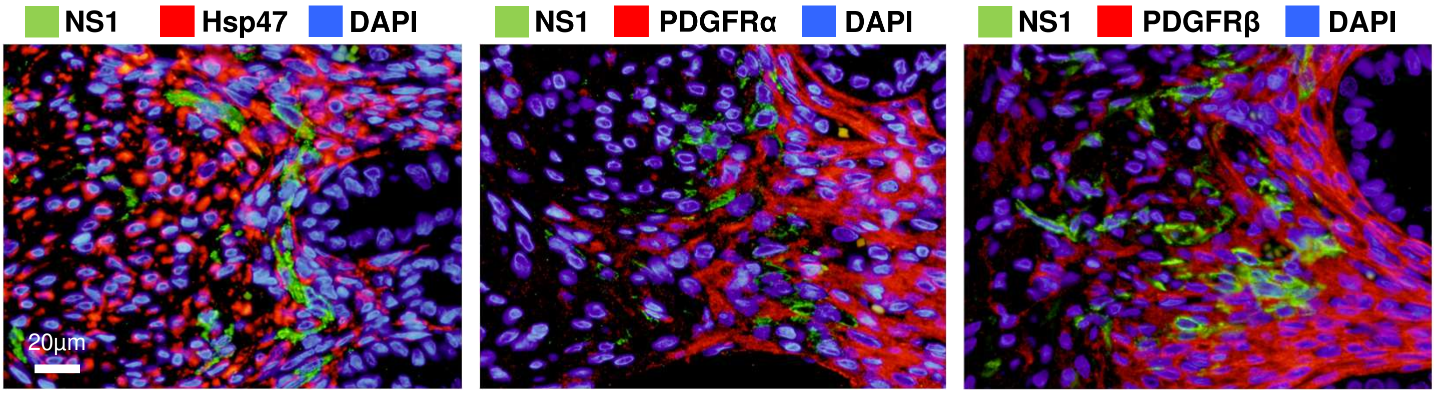

Figure S1 Double immunofluorescence staining of ZIKV NS1 and fibroblast markers in the BLE region. Uterine tissue from ZIKV-infected P-5 was stained with a combination of anti-NS1 antibody and an antibody against fibroblast marker such as Hsp47, PDGFRα or PDGFRβ. The viral NS1 protein and target tissue proteins are indicated by green and red fluorescence, respectively.
