# Supplementary material for: Pathological characterization of female reproductive organs prior to miscarriage induced by Zika virus infection in the pregnant common marmoset
Source: Microbiol Spectr. 2025 Feb 25;13(4):e02282-24. doi: 10.1128/spectrum.02282-24 (PMC11960083; doi:10.1128/spectrum.02282-24)
Supplement: Figure S2 — MPO staining with ovarian tissues. [file spectrum.02282-24-s0002.pdf]

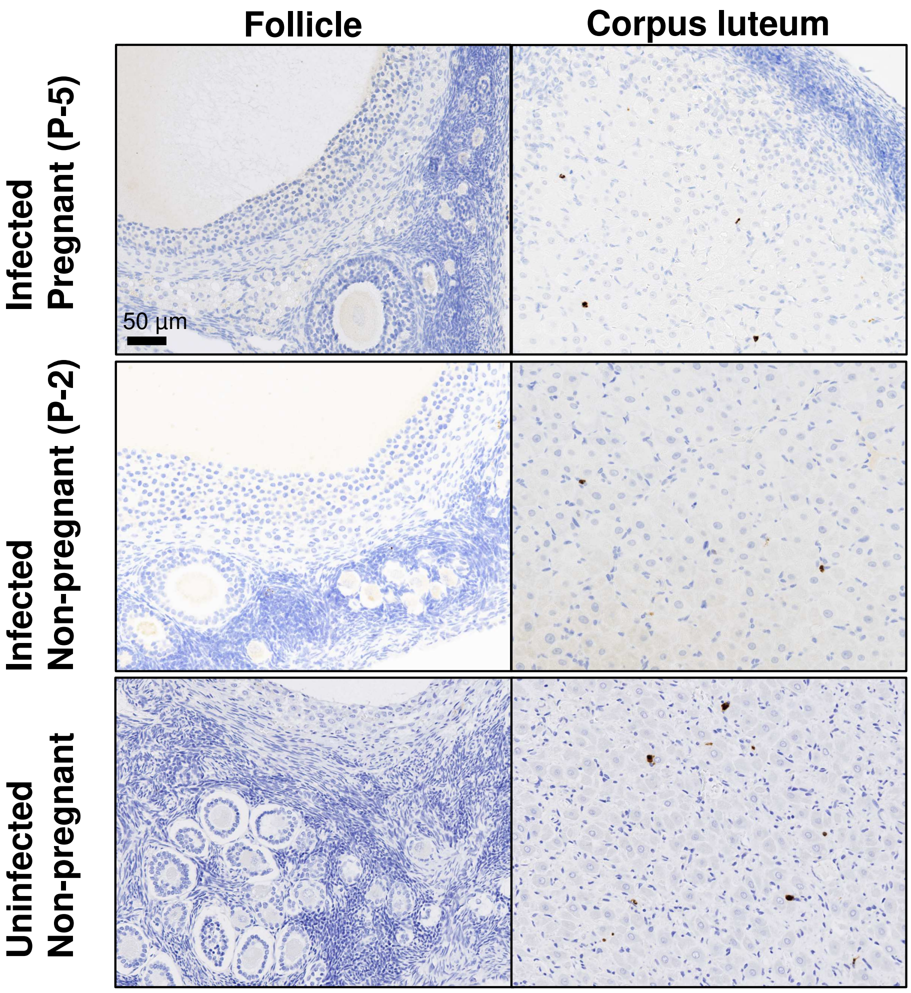

Figure S2 MPO staining with ovarian tissues. Ovary specimens derived from ZIKV-infected pregnant (P-5), ZIKV-infected non-pregnant (M-2) and uninfected non-pregnant marmosets were stained with anti-MPO antibody. The follicular and corpus luteum portions for each marmoset were shown.
