# Supplementary material for: Pathological characterization of female reproductive organs prior to miscarriage induced by Zika virus infection in the pregnant common marmoset
Source: Microbiol Spectr. 2025 Feb 25;13(4):e02282-24. doi: 10.1128/spectrum.02282-24 (PMC11960083; doi:10.1128/spectrum.02282-24)
Supplement: Figure S3 — Validation of specific antigen detection with anti-ZIKV NS1 antibody Mab 2-42 used in this study. [file spectrum.02282-24-s0003.pdf]

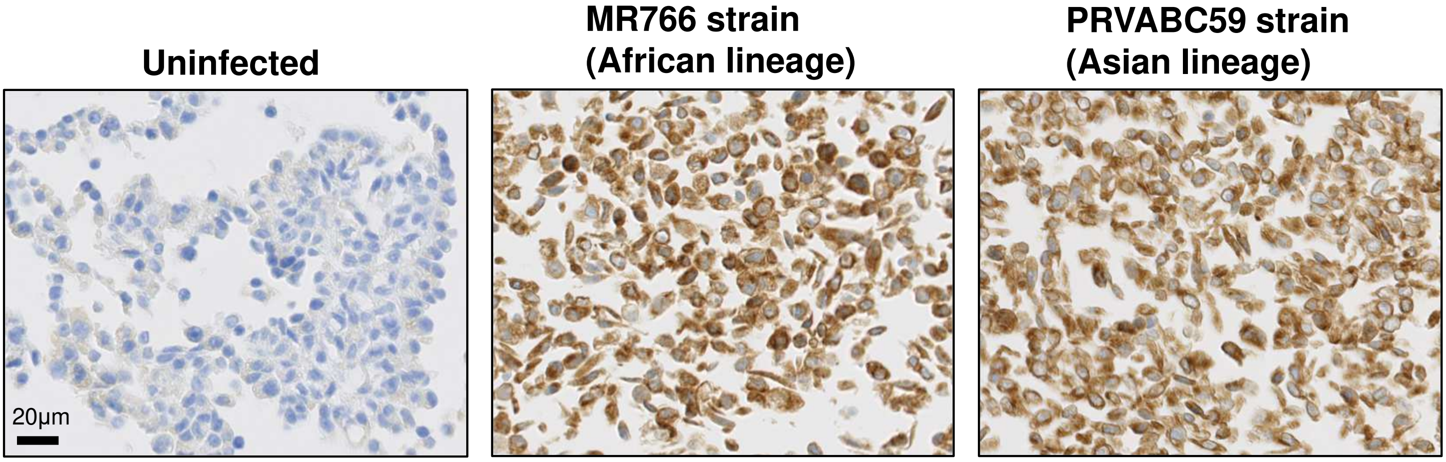

Figure S3 Validation of specific antigen detection with anti-ZIKV NS1 antibody Mab 2-42 used in this study. Vero cells derived from African green monkey kidney were infected with ZIKV; MR766 and PRVABC59 strains. Uninfected and ZIKV-infected cells were fixed with formalin at two days post infection. Paraffin-embedded sections were treated with the anti-NS1 antibody as well as 3, 3'-diaminobenzidine.
