# Supplementary material for: Pathological characterization of female reproductive organs prior to miscarriage induced by Zika virus infection in the pregnant common marmoset
Source: Microbiol Spectr. 2025 Feb 25;13(4):e02282-24. doi: 10.1128/spectrum.02282-24 (PMC11960083; doi:10.1128/spectrum.02282-24)
Supplement: Table S1 — Serum ZIKV RNA level of non-pregnant female and male marmosets. [file spectrum.02282-24-s0004.pdf]

Table S1: Serum ZIKV RNA level of non-pregnant female and male marmosets (copies/mL)

dpi	0	1	2	3	4	5	6	7	8
Non-pregnant female									
F-1	n.d.	5.1×10^4	n.t.	n.t.	1.1×10^4	n.t.	n.t.	n.t.	4.7×10^2
F-2	n.d.	1.9×10^5	n.t.	1.3×10^6	n.t.	n.t.	n.t.	2.1×10^2	n.t.
F-3	n.d.	3.5×10^7	n.t.	1.7×10^7	n.t.	3.9×10^5	n.t.	6.5×10^4	n.t.
F-4	n.d.	n.t.	9.9×10^5	n.t.	3.7×10^5	n.t.	6.5×10^3	3.2×10^3	n.t.
Male									
M-1	n.d.	1.9×10^4	n.t.	1.1×10^6	n.t.	8.0×10^2	65	n.t.	n.t.
M-2	n.d.	1.1×10^5	n.t.	7.6×10^6	n.t.	1.7×10^6	n.t.	3.7×10^4	n.t.

ZIKV RNA levels (copies/mL) in the sera of marmosets collected over time until day 8 post-infection (dpi) were determined by quantitative RT-PCR. The lower limit of quantitation for the ZIKV RNA assay system in this study was calculated to be 800 copies/mL. Measurements of low RNA levels below the limit of quantitation were extrapolated from the standard curves. n.d.: not detected, n.t.: not tested.
