# Supplementary material for: Pathological characterization of female reproductive organs prior to miscarriage induced by Zika virus infection in the pregnant common marmoset
Source: Microbiol Spectr. 2025 Feb 25;13(4):e02282-24. doi: 10.1128/spectrum.02282-24 (PMC11960083; doi:10.1128/spectrum.02282-24)
Supplement: Table S2 — Neutralizing antibody and IgM against ZIIKV in sera of infected marmosets. [file spectrum.02282-24-s0005.pdf]

Table S2: Neutralizing antibody and IgM against ZIIKV in sera of infected marmosets

Marmoset	Collection date	Neutralizing antibody (ID90)	Anti-ZIKV IgM
Female			
F-3	7 dpi	160	6.99
F-4	7 dpi	640	8.27
Male			
M-1	6 dpi	160	6.09

Neutralizing antibody and IgM against ZIKV were measured by SRIPs assay and ELISA. Neutralizing antibody titers were indicated by the serum dilution factor that inhibits ZIKV infection by 90% (ID90). Anti-ZIKV IgM titers were tested by ELSA and presented as ELISA indices, the ratio of absorbance of tested samples to the negative control. The ELISA index of 1.10 or higher is interpreted as positive.
